# Corporal Punishment in Schools and the Home

**Published:** 1902-01

**Authors:** Willis B. Parke

**Affiliations:** Atlanta, Ga.


### CORPORAL PUNISHMENT IN SCHOOLS AND THE HOME.*

BY WILLIS B. PARKE, M.D.,

ATLANTA, GA.

I do not think it is necessary nor even proper to discuss corporal punishment in the schools, since on investigation it is found that the best educators of our country, as well as other countries, have virtually settled that question, and of course the others will sooner or later follow.

But for the sake of fairness I will quote some of them to show that they have about decided the question. Professor Joseph Baldwin, author of Art School Management, Elementary Psychology

*Read before Georgia State Social Improvement Society, Atlanta, Ga., December 13-14, 1901.

and Psychology Applied to the Art of Teaching, says: "Corporal punishment is not educative," and for this reason, he says, it must go. At the beginning of the nineteenth century it was universal and popular, but at the close of the century it has not only become amazingly unpopular, but has virtually disappeared as a school punishment. He says it is not used in our colleges, nor in our high schools, nor in the first and second grades of our primary schools, nor our kindergartens. It is rarely used in the seventh and eighth grades of our grammar schools. In the four remaining grades it is becoming rarer and rarer. Some countries like France have abolished the rod. He says: "Public sentiment is getting strongly against the use of the rod. With the present century corporal punishment, it is believed, will utterly disappear from our schools." He says: "Our advancing civilization will not much longer tolerate the rod in our schools. The widest experience demonstrates that in our times corporal punishment hurts and does not help. The suffering inflicted does not even tend to work reformation, but it does tend to alienate pupils and parents." Dr. T. W. Harris says: "I think that better discipline can be obtained without the use of the rod than with it. I should not approve of even permitting corporal punishment in high schools." He says that it is better for the teacher to abolish corporal punishment than for the laws of the city to prohibit it unconditionally in the high schools. Col. F. W. Parker takes the extreme view. He says that corporal punishment is criminal. Reform and progress characterize civilization, and we somehow outgrow the antiquated. The old grows into the new. He also says that fear and dread, like a nightmare, depresses the pupil's effort. Often innocent pupils are condemned and punished on circumstantial evidence. Pupils are exceedingly sensitive to just and unjust punishment, which generates bitterness and resentment. To be just is the law of all schools, and must not be violated by the teacher. Prof. Baldwin sums as follows: "Public sentiment condemns the rod in our school. Corporal punishment has been abolished in the army, navy and penitentiary. Enlightened public opinion demands its discontinuance in our schools. Corporal punishment breeds trouble, even when parents consent to it; it hurts both pupil and teacher. It unfits the teacher for understanding the pupil and for governing through ennobling motives."

As the above are the exact quotations of some of the best educators of our country, I do not think it wise for me to further discuss corporal punishment in the schools, but will simply say, I fully agree with them. I take up the subject of home corporal punishment with some diffidence. To begin, I will balance Solomon's precept: "Spare the rod and spoil the child." With his example of plurality of wives, if you should insist on following his precepts, why not his example? The main object in this discussion is not to dictate to parents how they should control their own dear little ones, but to study the child's mind and their own peculiar characteristics as compared with those of adults, and after we have learned more of the great distinction between the child and the adult, and find that our treatment has been wrong, and our appreciation has been wanting, let us try to correct the almost unpardonable mistake. Prof. James M. Baldwin has made many very tedious experiments in studying the psychology of the child. He says: "In the first place the facts of the infant's own consciousness are very simple, that is, they are the child's sensations or memories simply, not his own observation of them. In the adult mind the disturbing influences of self-observation is a matter of notorious moment. It is impossible for me to report exactly what I feel, for the observation of it by my attention alters the character of how I feel. My volition also is a complex thing involving my personal pride and self-consciousness, but the child's emotion is as spontaneous as a spring." He says again: "In the study of the child's mind we have the advantage of simplicity on the physical side. We are able to take account of the physiological processes at a time when they are relatively simple. That is, before the nervous system has grown to maturity." For example: psychology used to hold that we have a speech, an inborn mental endowment, which is capable of further analysis, but support of this position is wanting when we turn to the brain of the infant. Not only do we fail to find the series of centers now known to be the "speech zone," but even those we do find have not yet taken up the function of speech, either alone or together. In other words, the primary object of speech of the various centers involved is not speech, but some other simpler function; and speech arises by development from a union of these separate functions. It can be easily seen by these observations and experiments of the psychologists that the child is not only simple in its mental actions as well as spontaneous, but the physiological development is the result of the uniting of the one or more of the simple brain actions.

Hence one of the great errors parents make in deciding the right and wrong of their children's actions. They interpret their motives and actions as they would their own, or the matured adult, not taking into consideration the child's act being simple and not complex.

To illustrate: I knew an instance where a mother whipped her little daughter, three years old, for sending her word by a servant, who was sent after the child from a neighbor's near by, that she would not come home with the servant, but would come with her mother. The mother misconstrued the motive of the child, for on close questioning the little condemned sinner it was found the child had one motive; not impudence, malice aforethought, as the mother had judged, but she did not like the new servant, who had no doubt mistreated the little innocent one. I will say in justice to children that when corporal punishment is inflicted unjustly, a great wrong is done that time may never heal; like the mighty oak that stands in the forest bidding defiance to the storm that may come upon it, yet when it was only a small bush, perchance, a slight injury was made on its external bark, and kind nature healed over the surface injury, but as the tree grew on to maturity, that seemingly slight surface injury ate its way into the very heart of the mighty oak, and the storm came upon it and on account of its former injury it swayed and fell by the wayside; so it might be with that precious one that God gave you to direct in the right way, when an unjust punishment or injury was inflicted because you did not understand the child's simple way. The external injury was soon healed over, but the effect reached the heart, and when the trials came upon him in mature life, in a fit of anger caused him to thrust the deadly dagger into his fellow man and he became a murderer. We cannot impress too strongly the probable effect of the injury to the closely allied physical and moral nature of childhood. Again, psychologists are better equipped to make observations of the child and their own peculiarities than parents, "for a true psychologist will follow one trait from its origin to its destiny, closely noting cause and effect, while the parent will make disconnected observations without regard to cause and effect." Parents are liable to come to wrong conclusions in regard to physical conditions of their children. To illustrate: A mother asked the family physician in the presence of her little daughter what would be the best way to break a child from twitching its face and blinking its eyes as a bad habit. The physician looked the little daughter in the face, and, to the astonishment of the mother, says: "Your little daughter has the first symptoms of Saint Vitus' Dance." No doubt that child, to its mortification, had received many reproofs for the bad habit.

Psychologists have made a very comprehensive distinction in children into the two great spheres of reception and action, or the "sensory and motory;" the motory or active child is very responsive to "suggestion." "He also gets his impressions through the memory of action. He remembers a thing as how it acted, or how he acted when he learned it. Such children learn very readily, for they are on the move; they observe quickly, and by parents and teachers are called smart to learn; but to their disappointment they find this smart motery child just as quick to forget. They do not take into consideration that the little, active, receptive brain has taken in more than it can possibly assimilate." Such children receive their share of corporal punishment for forgetting their lessons and what was plainly told them. Morally the child was not to blame. Psychologists say a motery child should not be encouraged in observing everything, nor pushed in their duties or lessons, for, like a spirited horse, they will do more than their capacity will admit. "Such children are dominated by habit, and this means that his nervous system by undue predominance of certain elements in his education sets quickly in the direction of motery discharge. The great channels of readiest outpouring from the brain into the muscles have become fixed. They tend to unstable equilibrium in the direction of certain motory combinations, which in their turn represent certain classes of acts. This is habit, and the child of extreme motor type is always a creature of habit." A child of extreme motory type cannot be expected to be benefited by corporal punishment, for as said, "they are creatures of habit, or automatic action." "They should not be allowed to repeat themselves; they should be taught something new, making progress, for habit cannot exist in progress." "In this connection great care should be exercised in the suggestion, for it is well established that a suggestion not to do a thing has no negative force, but on the contrary in the early period, it amounts only to a stronger suggestion in the positive, since it adds emphasis to the thing which is forbidden. Indeed, the philosophy of all punishments rests on this consideration: that is, that unless the penalty tends to fill the mind with some object other than the act punished, it does more harm than good."

"The sensory children who receive their impressions mostly through the nerves of sight and hearing are passive; more troubled with physical inertia, more contemplative when a little older, less apt in learning to act out new movements, less quick to take the hint, etc. These children are generally further distinguished as being much less suggestionable. They seem duller when young. The motory child will show sorrow by loud crying and vigorous actions while the misunderstood sensory child will grieve in quiet, and continue to grieve when the motory child has forgotten the disagreeable occurrence altogether." The sensory child is usually dubbed dull, slow to learn or comprehend and is known and often called by teachers, and even parents, "blockhead," "dunce," etc. The sensory child tends to be timid in the presence of strangers and the unknown and uncertain to learn from, one or few experiences, and to hold back until satisfactory assurance that danger is absent. Psychologists say "the treatment of the sensory child is a problem of even greater difficulty than that of his motory brother. We have no way to read thought directly, so just in so far as the sensory child is less active to that degree he is less impressive, less revealing. Show him his mistake with the utmost deliberation and kindness. In arranging children's games see that the timid, sensory child gets an active part. Cultivate the imitative tendency in him, never allowing him to think he has failed or even made a mistake, and if he should be punished for not successfully competing with the motory child it would be very disastrous." It is usually said by most parents that they have not the time to devote to the whims of their children. Psychologists say that with the aid of experiments and scientific research that these peculiarities are not whims, but are inborn traits and tendencies, that are more or less aggravated by environments and improper education. If this be true, a great responsibility devolves upon the parent that cannot be dismissed with a trivial excuse. Let it be understood that all the types and tendencies of children as pointed out in this paper are not to be considered imperfections, but the tendencies unguided, the sensory child misunderstood, the motory boy lashed into obedience, result not merely as imperfections, but oftentimes culminate in sins of the deepest dye.

It seems then that it is not the duty of parents to coerce their God-given children with the iron-rod of "rule or ruin," but to adjust themselves to the great problem that confronts them by overcoming their own sins and imperfections, for it is said, "except ye be converted, and become as little children, ye shall not enter into the Kingdom of Heaven." And again: "Suffer little children to come unto me and forbid them not, for of such is the Kingdom of Heaven."

